# ASSISTED LIVING ADMINISTRATORS’ PERSPECTIVES OF HOW COVID-19 AFFECTED DIRECT CARE STAFF

**DOI:** 10.1093/geroni/igad104.2046

**Published:** 2023-12-21

**Authors:** Carlyn Vogel, Debra Dobbs, Lindsay Peterson, Victor Molinari, Hongdao Meng

**Affiliations:** University of South Florida, Tampa, Florida, United States; University of South Florida, Tampa, Florida, United States; University of South Florida, Tampa, Florida, United States; University of South Florida, Tampa, Florida, United States; University of South Florida, Tampa, Florida, United States

## Abstract

**Background:**

Coronavirus disease 2019 (COVID-19) presented many new challenges within assisted living communities (ALCs) and exacerbated preexisting challenges. With high quality of care in ALCs as the continuous goal to achieve, it is important to recognize challenges faced in these communities, especially those related to staffing and COVID-19. Objective: The objective was to investigate issues related to staffing and the effect of COVID-19 in ALCs, including staff absence, leadership, impacts on staff, and impacts on staff retention.

**Methods:**

Participants were ALC administrators (N=129) from the parent study of the Strategic Approach to Facilitating Evacuation by Health Assessment of Vulnerable Elderly in Nursing Homes II (SAFEHAVEN II) funded by the National Institute on Aging. Quantitative analysis was used to determine structure and process characteristics of ALCs during COVID-19 that impacted a process outcome. Qualitative analyses were used to gain perspectives of administrators about the impact of COVID-19 within their ALCs.

**Results:**

Provision of memory care services (structure characteristic), challenge with staff sent home to comply with COVID-19 precautions, challenge paying staff for time off due to COVID-19, and staff anxiety (process characteristics) were significant predictors of staff absence from work (process outcome). Three key themes interpreted from administrators’ interviews were Leadership Shown Toward Staff, Impact of COVID-19 on Staff, and Impact of COVID-19 on Staff Retention. Implications: This dissertation discusses implications for research, policy, and practice to inform continual efforts to improve aspects of staffing and the quality of resident care in ALCs.

